# MIC19 Exerts Neuroprotective Role via Maintaining the Mitochondrial Structure in a Rat Model of Intracerebral Hemorrhage

**DOI:** 10.3390/ijms241411553

**Published:** 2023-07-17

**Authors:** Siyuan Yang, Xulong Yin, Jiahe Wang, Haiying Li, Haitao Shen, Qing Sun, Xiang Li

**Affiliations:** 1 Department of Neurosurgery & Brain and Nerve Research Laboratory, The First Affiliated Hospital of Soochow University, Suzhou 215006, China; 2 Institute of Stroke Research, Soochow University, Suzhou 215006, China; 3 Department of Neurology, The First Affiliated Hospital of Soochow University, Suzhou 215006, China

**Keywords:** intracerebral hemorrhage, MICOS, MIC19, mitochondria

## Abstract

As an essential constituent of the mitochondrial contact site and cristae organization system (MICOS), MIC19 plays a crucial role in maintaining the stability of mitochondrial function and microstructure. However, the mechanisms and functions of MIC19 in intracerebral hemorrhage (ICH) remain unknown and need to be investigated. Sprague Dawley (SD) rats injected with autologous blood obtained from the caudal artery, and cultured neurons exposed to oxygen hemoglobin (OxyHb) were used to establish and emulate the ICH model in vivo and in vitro. Lentiviral vector encoding MIC19 or MIC19 short hairpin ribonucleic acid (shRNA) was constructed and administered to rats by intracerebroventricular injection to overexpress or knock down MIC19, respectively. First, MIC19 protein levels were increased after ICH modeling. After virus transfection and subsequent ICH modeling, we observed that overexpression of MIC19 could mitigate cell apoptosis and neuronal death, as well as abnormalities in mitochondrial structure and function, oxidative stress within mitochondria, and neurobehavioral deficits in rats following ICH. Conversely, knockdown of MIC19 had the opposite effect. Moreover, we found that the connection between MIC19 and SAM50 was disrupted after ICH, which may be a reason for the impairment of the mitochondrial structure after ICH. In conclusion, MIC19 exerts a protective role in the subsequent injury induced by ICH. The investigation of MIC19 may offer clinicians novel therapeutic insights for patients afflicted with ICH.

## 1. Introduction

Intracerebral hemorrhage (ICH) is considered as a devastating disease, accounting for 15–20% of all strokes worldwide, and has high rates of fatality and morbidity. It can cause serious financial and psychological burdens for ICH patients’ families [1,2]. ICH generally emerges as a rapid expansion of hematoma in the brain tissues which may expand into the ventricular system and subarachnoid spaces [3,4]. Despite recent progress in clinical therapy, clinical outcomes of ICH patients still remain unsatisfactory owing to primary and secondary brain injury [5,6,7]. Secondary brain injury (SBI) is reported to be caused by the elevated intracranial pressure (ICP) owing to the stimulation and compression of hematoma in brain, and its mechanisms mainly include brain edema, oxidative stress, necrotic cell death, mitochondrial damage, and inflammatory factors secretion [8,9,10,11,12]. Thus, the investigation of nerve protection in ICH-induced SBI is needed.

Mitochondria are an indispensable microstructure in cells and perform critical functions such as energy generation, antioxidant stress, modulation of cell proliferation, biosynthesis of certain substances, and other functions [13,14]. Moreover, mitochondrial damage and mitochondrial dysfunction are considered as critical factors related to the subsequent injury induced by ICH [15,16]. However, the role of mitochondrial dysfunction in ICH-induced brain injury remains unclear.

As the core elements of the mitochondrial contact site and cristae organization system (MICOS), MIC19 and MIC60 play positive roles in maintaining the stability of the normal function and microstructure of mitochondria [17,18]. Recently, a study reported that MIC19 can directly interact with inner membrane protein MIC60 and outer membrane protein SAM50 in mitochondria to form the SAM50–MIC19–MIC60 axis, and the existence of this axis is reported to be indispensable for the stability of the mitochondrial structure and functions [18,19,20]. As reported, MIC19 also takes part in regulating protein transmembrane transport in mitochondria, energy generation, apoptosis, and other physiological processes [17].

In this experiment, autologous blood of rats in vivo and oxygen hemoglobin (OxyHb) in vitro were used to simulate the occurrence of ICH. The application of non-heparinized autologous blood in the autologous blood injection method is closer to human cerebral hemorrhage. It is suitable for studying the natural process and pathological morphological characteristics of ICH. However, it may not fully mimic the occurrence of ICH due to the microvascular breakdown in the human condition [21]. Therefore, we also used OxyHb to mimic ICH in vitro. The method can mimic the occurrence of ICH in vitro. However, blood from microvascular rupture also contains other components in addition to OxyHb, such as inflammatory factors, plasmin, platelets, etc. [22].

Hence, it is critical for us to conduct research to reveal the potential effects and mechanisms of MIC19 during the occurrence of ICH. The investigation of MIC19 may expand the understanding of mechanisms in ICH and might provide clinicians with innovative therapeutic thoughts for patients suffering from ICH.

## 2. Results

### 2.1. General Observations

**Figure 1:**
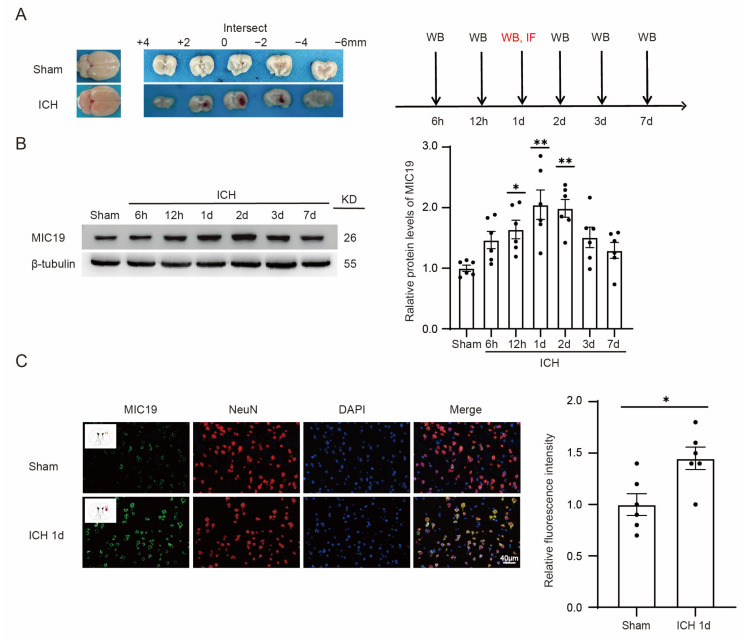
The protein level of MIC19 increased in the brain after ICH in rats. (**A**) Representative coronal brain sections (2 mm) in sham and ICH groups; 0 means the location of the injection point. (**B**) Western blot analysis and quantification of MIC19 protein levels in the perihematomal region after ICH (* *p* < 0.05, 12 h: *p* = 0.033, ** *p* < 0.01, 1 d: *p* = 0.0002, ** *p* < 0.01, 2 d: *p* = 0.0005 vs. sham group, n = 6, ANOVA type: ordinary one-way ANOVA). (**C**) Double immunofluorescent analysis was performed with MIC19 antibodies (green), neuronal marker NeuN (red) in brain sections. Nuclei were fluorescently labeled with 4,6-diamino-2-phenylindole DAPI (blue) (* *p* < 0.05, 1 d: *p* = 0.0144 vs. sham group, n = 6), Representative images of the sham and ICH (1 d) groups are shown. Scale bar = 40 μm, magnification: 40× mirror. Mean ± SE was used to describe all data, and mean values in the sham group were normalized to 1.0.

There was no significant difference in body weight, room temperature, or other basic situations in the experimental group compared with in the sham group. As shown in the Supplementary Materials, the mortality rate of rats was 2.94% (1/40 rats) in the sham group, while the rate was 12.7% (31/243 rats) in the ICH group. Representative brain sections in the sham and ICH groups are presented in Figure 1A.

### 2.2. MIC19 Expression Increases in Brain Tissues and Primary-Cultured Cortical Neurons after ICH Induction

At first, a significant increase in MIC19 protein levels was found at 24 h after ICH modeling in vivo via Western blot (Figure 1B). Next, immunofluorescent staining with MIC19, NeuN (a marker specific to neurons), and 4,6-diamino-2-phenylindole (DAPI) (a marker specific to nuclei) was performed to assess the expression and cellular distribution of MIC19 protein in rats before and after ICH. Immunofluorescence quantitative analysis indicated that the protein level of MIC19 was upregulated one day after ICH, and the cellular distribution of MIC19 was in the cytoplasm, as shown in Figure 1C.

### 2.3. MIC19 Overexpression Alleviates ICH-Induced Neurodegeneration and Neuronal Apoptosis in Rats

**Figure 2:**
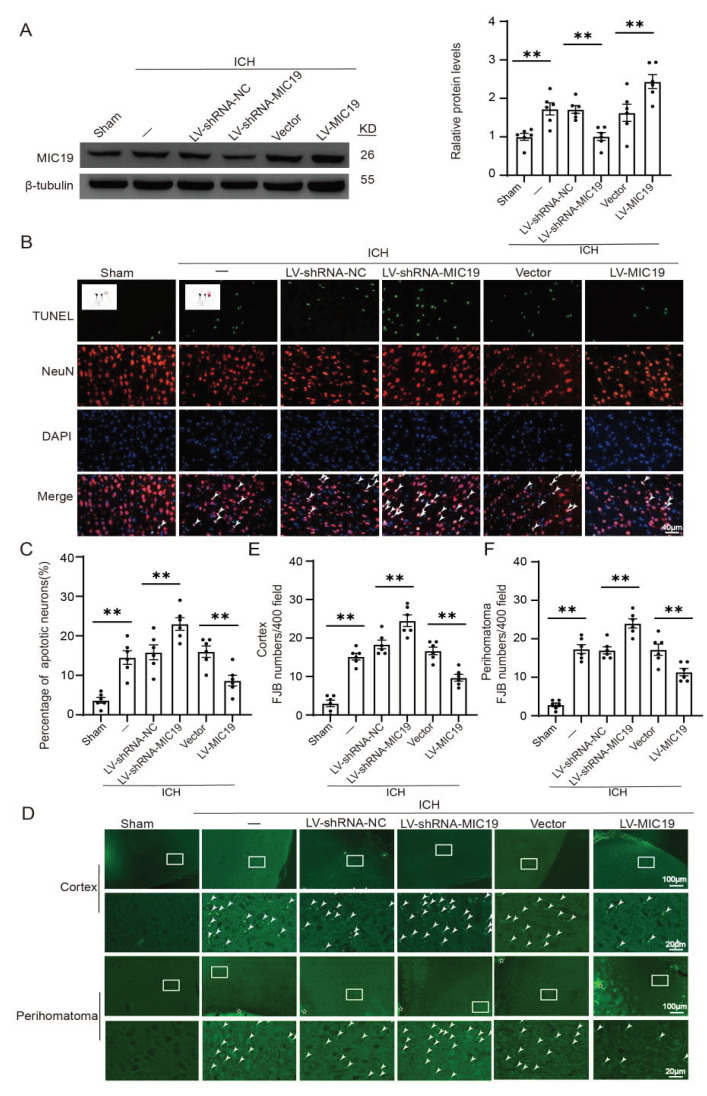
MIC19 overexpression mitigated ICH-induced neuronal apoptosis and neurodegeneration in rats via virus transfection. (**A**) Western blot analysis and quantification of the effects of MIC19 siRNA and overexpression plasmids (n = 6) (** *p* < 0.01 vs. sham: *p* = 0.0056; ** *p* < 0.01, LV-shRNA-NC vs. LV-shRNA-MIC19: *p* = 0.0078; ** *p* < 0.01, vector vs. LV-MIC19: *p* = 0.0019; n = 6, ANOVA type: ordinary one-way ANOVA). (**B**,**C**) Double staining for terminal deoxynucleotidyl-transferase-mediated dUTP nick end labeling (TUNEL) (green), neuronal marker NeuN (red) and DAPI (blue); arrows point to TUNEL-positive neurons (** *p* < 0.01 vs. sham: *p* < 0.0001; ** *p* < 0.01, LV-shRNA-NC vs. LV-shRNA-MIC19: *p* = 0.0058; ** *p* < 0.01, vector vs. LV-MIC19: *p* = 0.0047; n = 6, ANOVA type: ordinary one-way ANOVA). Scale bar = 40 μm, magnification: 40× mirror. (**D**–**F**) Fluoro-jade B (FJB) staining in cortex and perihematoma region is shown; arrows point to FJB-positive cells (cortex: ** *p* < 0.01 vs. sham: *p* < 0.0001; ** *p* < 0.01, LV-shRNA-NC vs. LV-shRNA-MIC19: *p* = 0.0008; ** *p* < 0.01, vector vs. LV-MIC19: *p* = 0.0002; n = 6; perihematoma: ** *p* < 0.01 vs. sham: *p* < 0.0001; ** *p* < 0.01, LV-shRNA-NC vs. LV-shRNA-MIC19: *p* = 0.0002; ** *p* < 0.01, vector vs. LV-MIC19: *p* = 0.0016; n = 6, ANOVA type: ordinary one-way ANOVA, hematoma was pointed with star). Counts of FJB-positive cells in the cortex and perihematoma region are shown. Scale bar = 100 μm, magnification: 10× mirror. Mean ± SE was used to describe all data, and mean values in the sham group were normalized to 1.0.

In order to investigate the effects of MIC19 on ICH, the protein levels of MIC19 were knocked down and overexpressed by administering the lentiviral vector and their control agents to rats. Next, all grouped rats were used for ICH modeling 7 days after virus administration, and the samples were collected 1 d after ICH in vivo due to a significant increase in MIC19 at 24 h after ICH. Then, Western blot analysis and the follow-up chart were used to confirm the effects of MIC19 siRNA transfection and MIC19 overexpression plasmid transfection (Figure 2A).

FJB and TUNEL staining were conducted to research the effect of MIC19 on the death of neurons during ICH. In TUNEL staining, there was more neuronal apoptosis after ICH treatment. Furthermore, the TUNEL-positive rate of neurons was found to decrease after MIC19 overexpression and increased after the knockdown of MIC19 (Figure 2B,C). After ICH induction, the FJB staining showed more FJB-positive neurons in the cortex tissue and the perihematoma tissue. The number of FJB-positive neurons was greater after MIC19 knockdown. On the contrary, the reaction was reversed, and the number of FJB-positive neurons decreased after the overexpression of MIC19 (Figure 2D,E). The phenomenon was generally consistent with the trend in the experiment of TUNEL staining.

### 2.4. Overexpression of MIC19 Reverses ICH-Induced Mitochondrial Damage in Rats

**Figure 3:**
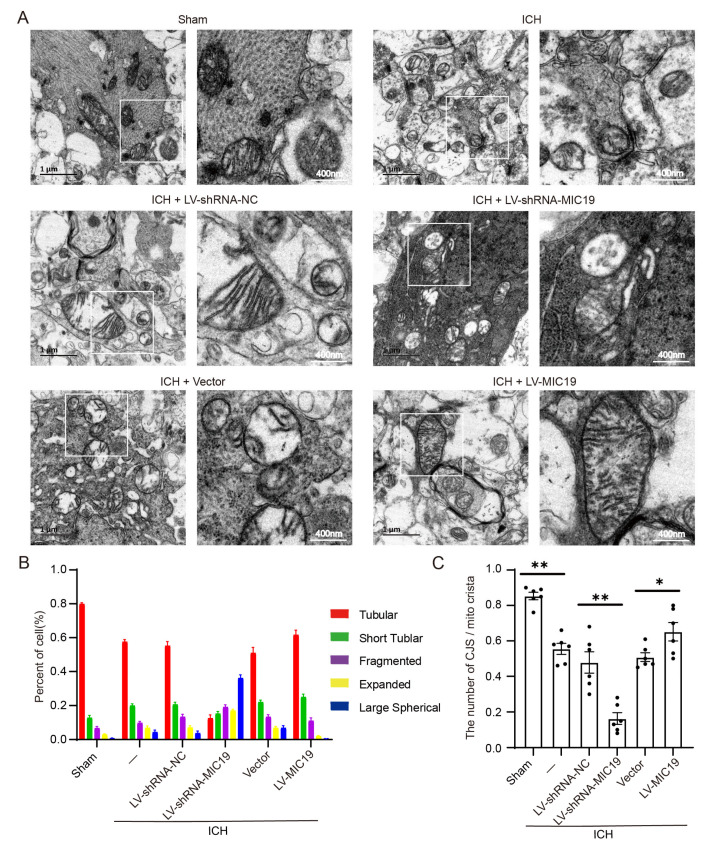
Effects of knockdown and overexpression of MIC19 on ICH-induced mitochondrial structure destruction in rats. (**A**) Mitochondrial morphologies under different conditions. Sham group: relatively intact mitochondrial membrane structure; ICH group, ICH + vector group, and ICH + LV-shRNA-NC group: inner membrane collapse, CJs; ICH + LV-MIC19 group: mitochondrial structural; ICH + LV-shRNA-MIC19 group: mitochondrial damage increased. Scale bar = 400 nm. (**B**) Statistical analysis of the proportion of different structures of crista in cells surrounding the hematoma (tubular crista: ** *p* < 0.01 vs. sham: *p* < 0.0001; ** *p* < 0.01, LV-shRNA-NC vs. LV-shRNA-MIC19: *p* < 0.0001; ** *p* < 0.01, vector vs. LV-MIC19: *p* = 0.0004; n = 6, ANOVA type: two-way ANOVA). (**C**) Number of cristea junctions (CJs)/mito-cristea in different groups (** *p* < 0.01 vs. sham: *p* < 0.0001; ** *p* < 0.01, LV-shRNA-NC vs. LV-shRNA-MIC19: *p* < 0.0001; * *p* < 0.05, vector vs. LV-MIC19: *p* = 0.0465; n = 6, ANOVA type: ordinary one-way ANOVA). Mean ± SE was used to describe all data, and mean values in the sham group were normalized to 1.0.

Electron microscopy was utilized to analyze the changes in the morphology and microstructure of mitochondria in rats. In the study, mitochondrial cristae junctions were found to be lost after ICH modeling. Some mitochondria in the ICH + LV-shRNA-MIC19 rats exhibited a swollen morphology and even ruptured the outer mitochondrial membrane. In contrast to in the ICH + LV-shRNA-NC group, mitochondria were found to be damaged more seriously in the ICH + LV-shRNA-MIC19 group. However, compared with in the ICH + vector group, the situation of the mitochondrial structure was better in the ICH + LV-MIC19 group (Figure 3A). Mitochondrial morphology was divided into five classifications (tubular, short tubular, fragmented, expanded, and large spherical) and quantified according to the related literature. The five types of cristae in mitochondria were counted and recorded in different interventional groups (Figure 3B). The study found that the percentage of tubular cristae decreased significantly after ICH modeling. The rate of tubular cristae was again increased after MIC19 overexpression. However, the percentage of tubular cristae tended to decrease after MIC19 knockdown. In Figure 3C, the percentage of cristae junctions (CJs)/mito-cristae increased after ICH modeling, and knockdown of MIC19 worsened the effect compared with that in the ICH + LV-shRNA-NC group.

### 2.5. Overexpression of MIC19 Improves Behavioral Deficits in Rats after ICH Induction

**Figure 4:**
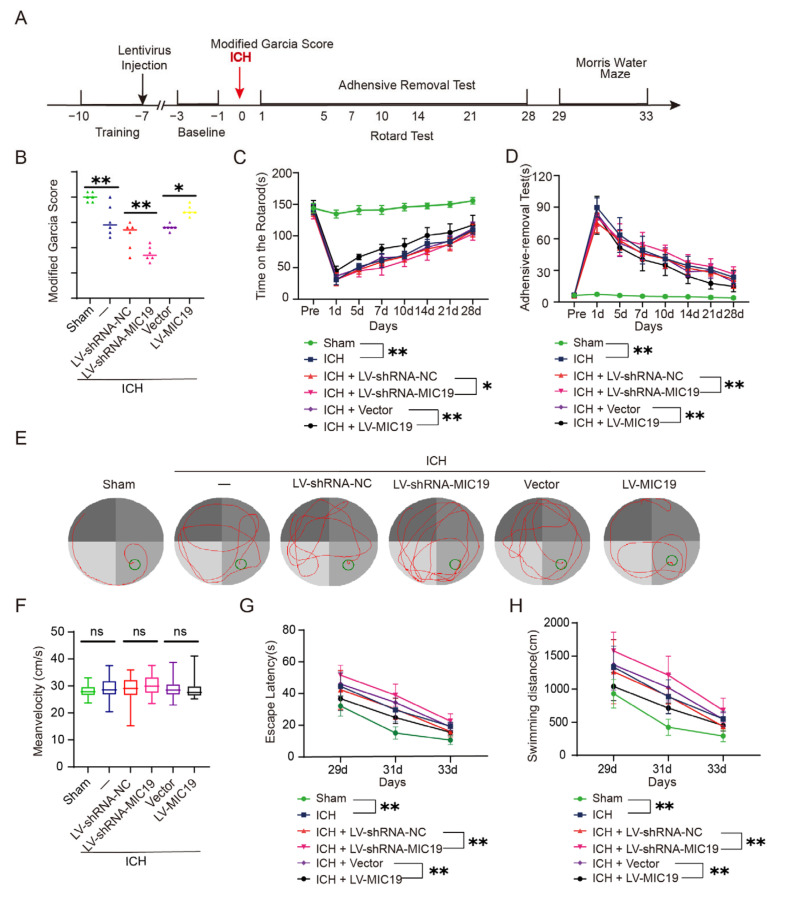
Effects of knockdown and overexpression of MIC19 on behavioral changes in ICH rats. (**A**) Flow chart of the procedure of behavior tests. (**B**) Neurological behavioral scores (** *p* < 0.01 vs. sham: *p* = 0.0005; ** *p* < 0.01, LV-shRNA-NC vs. LV-shRNA-MIC19: *p* = 0.0094; * *p* < 0.05, vector vs. LV-MIC19: *p* = 0.0139; n = 6, ANOVA type: two-way ANOVA). (**C**) Results of rotarod test (** *p* < 0.01 vs. sham: *p* < 0.0001; * *p* < 0.05, LV-shRNA-NC vs. LV-shRNA-MIC19: *p* = 0.0342; ** *p* < 0.01, vector vs. LV-MIC19: *p* < 0.0001; n = 10, ANOVA type: two-way ANOVA). (**D**) Results of adhesive removal test (** *p* < 0.01 vs. sham: *p* < 0.0001; ** *p* < 0.01, LV-shRNA-NC vs. LV-shRNA-MIC19: *p* = 0.0017; ** *p* < 0.01, vector vs. LV-MIC19: *p* = 0.0010; n = 10, ANOVA type: two-way ANOVA). (**E**) Representative images of the Morris water maze are shown., the red lines showed the trajectory of rats, the green circles showed the underwater platform. (**F**) The mean velocity for rats swimming in the pool was analyzed (ns, no significant difference). (**G**) The escape latency for rats to arrive at the final destination at 29, 31, and 33 days after ICH was analyzed (** *p* < 0.01 vs. sham: *p* < 0.0001; ** *p* < 0.01, LV-shRNA-NC vs. LV-shRNA-MIC19: *p* < 0.0001; ** *p* < 0.01, vector vs. LV-MIC19: *p* = 0.001; n = 10, ANOVA type: two-way ANOVA). (**H**) The swimming distance for rats to arrive at the underwater platform at 29, 31, and 33 days after ICH was analyzed (** *p* < 0.01 vs. sham: *p* < 0.0001; ** *p* < 0.01, LV-shRNA-NC vs. LV-shRNA-MIC19: *p* < 0.0001; ** *p* < 0.01, vector vs. LV-MIC19: *p* = 0.0001; n = 10, ANOVA type: two-way ANOVA). The behavioral data were reported as the mean ± SD.

We further examined whether modulation of MIC19 expression levels had an effect on the neurological deficit of ICH rats. In Figure 4A, a flow chart is shown to elaborate on the procedure of the behavior tests. The neurological scores of the rats became worse after ICH modeling, and MIC19 overexpression significantly improved the neurological scores, which means MIC19 overexpression reduced the neurological damage induced by ICH (Figure 4B). In the rotarod test, the rats in the ICH + LV-MIC19 group had the ability to run in the rod with more time, and the rats in the ICH + LV-shRNA-MIC19 group behaved worse than those in the ICH + LV-shRNA-NC group, which means that overexpression of MIC19 improved the locomotor functions and coordinating ability of ICH rats (Figure 4C). Additionally, overexpression of MIC19 alleviated sensory and motor dysfunction of rats undergoing ICH, while knockdown of MIC19 had the opposite effects in the adhesive removal test (Figure 4D). Finally, we used the Morris water maze test, conducted 29, 31 and 33 days post ICH, to research the long-term effect of overexpression and knockdown of MIC19 on cognitive impairment induced by ICH (Figure 4E). There was no significant difference in mean velocity between the six interventional groups, which eliminated the test’s possible influence factor due to behavioral impairment (Figure 4F). The Morris water maze was used to research the time and length that these rats took to find the platform in the maze and to evaluate the rats’ learning, memory, and cognition ability. After ICH, the swimming distance and escape latency for rats to arrive at the underwater platform in the maze were significantly longer than those in the sham group. Furthermore, the ICH + LV-MIC19 group tended to behave better, and the rats’ dysfunction in learning, memory, and cognition was alleviated, whereas those of the ICH + LV-shRNA-MIC19 group behaved worse (Figure 4G,H).

### 2.6. Overexpression of MIC19 Inhabits Oxidative Stress Induced by ICH and Enhances Cytochrome c Oxidase Activity in Rats

**Figure 5:**
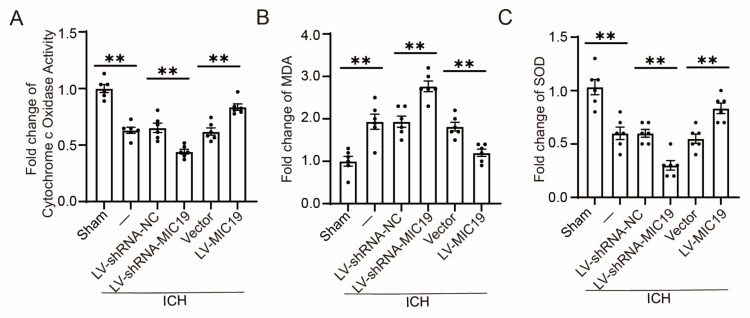
Effects of knockdown and overexpression of MIC19 on oxidative stress and cytochrome c oxidase activity in rats. (**A**) Cytochrome c Oxidase activity is shown (** *p* < 0.01 vs. sham: *p* < 0.0001; ** *p* < 0.01, LV-shRNA-NC vs. LV-shRNA-MIC19: *p* = 0.0002; ** *p* < 0.01, vector vs. LV-MIC19: *p* = 0.0001; n = 6, ANOVA type: ordinary one-way ANOVA). (**B**) The content of MDA is shown (** *p* < 0.01 vs. sham: *p* < 0.0001; ** *p* < 0.01, LV-shRNA-NC vs. LV-shRNA-MIC19: *p* = 0.0002; ** *p* < 0.01, vector vs. LV-MIC19: *p* = 0.0052; n = 6, ANOVA type: ordinary one-way ANOVA). (**C**) The content of SOD is shown (** *p* < 0.01 vs. sham: *p* < 0.0001; ** *p* < 0.01, LV-shRNA-NC vs. LV-shRNA-MIC19: *p* = 0.0009; ** *p* < 0.01, vector vs. LV-MIC19: *p* = 0.0016; n = 6, ANOVA type: ordinary one-way ANOVA). Mean ± SE was used to describe all data, and mean values in the sham group were normalized to 1.0.

We used the Cytochrome c Oxidase Activity Colorimetric Assay Kit to detect the Cytochrome c Oxidase activity among these six interventional groups. After being induced by ICH, lower activity of Cytochrome c Oxidase was shown in rats. The effect improved in the ICH + LV-MIC19 group and worsened in the MIC19 knockdown group (Figure 5A). We found that the level of malondialdehyde (MDA) reflecting the lipid peroxidation increased after ICH induction in the rats’ brain tissues, and the MDA level became worse in the MIC19 knockdown group. Furthermore, the content of superoxide dismutase (SOD), which can eliminate oxygen free radicals, decreased after ICH induction, and this effect improved in the MIC19 overexpression group (Figure 5B,C).

### 2.7. Overexpression of MIC19 Exerts Neuroprotective Effects in Neurons

**Figure 6:**
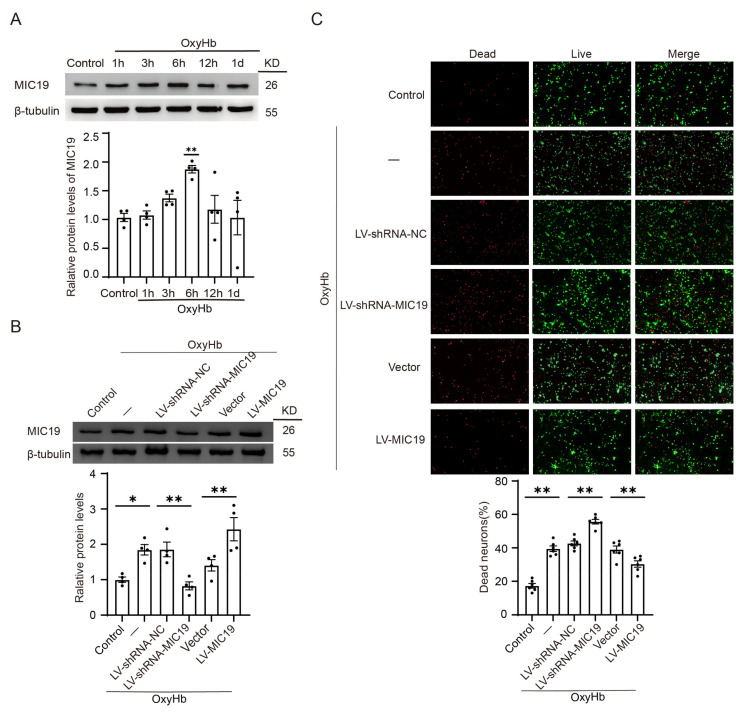
ICH increased MIC19 protein levels in neurons, and MIC19 rescued ICH-induced neuronal cell death. (**A**) Western blot analysis for the changes in MIC19 protein levels at 1 h, 3 h, 6 h, 12 h, and 1 d in cultured neurons (** *p* < 0.01, 6 h: *p* = 0.0096 vs. sham group, n = 4, ANOVA type: ordinary one-way ANOVA). (**B**) Western blot analysis of the effects of overexpression and knockdown of MIC19 in cultured neurons (* *p* < 0.05 vs. sham: *p* = 0.0168; ** *p* < 0.01, LV-shRNA-NC vs. LV-shRNA-MIC19: *p* = 0.0037; ** *p* < 0.01, vector vs. LV-MIC19: *p* = 0.0039; n = 4, ANOVA type: ordinary one-way ANOVA). (**C**) Live–dead cell staining was utilized to assess the cell apoptosis: live cells (green) and dead cells (red) (** *p* < 0.01 vs. sham: *p* < 0.0001; ** *p* < 0.01, LV-shRNA-NC vs. LV-shRNA-MIC19: *p* < 0.0001; ** *p* < 0.01, vector vs. LV-MIC19: *p* = 0.0028; n = 6, ANOVA type: ordinary one-way ANOVA). Scale bar = 100 μm, magnification: 10× mirror. Mean ± SE was used to describe all data, and mean values in the sham group were normalized to 1.0.

Next, we conducted our study in vitro and investigated the role of MIC19 in neurons handled by OxyHb. A significant increase in MIC19 protein levels was also found 6 h after being treated by oxygen hemoglobin (OxyHb) in vitro via Western blot (Figure 6A). Thus, the time point of sample collection after virus transfection was 6 h after ICH modeling in vitro. We also overexpressed or knocked down the expression of MIC19 via virus transfection (Figure 6B). In live–dead cell staining, more neurons were found to be dead (red) after being treated by OxyHb. Furthermore, the percentage of dead cells in this experiment was increased after MIC19 knockdown, while it was reversed after MIC19 overexpression (Figure 6C).

### 2.8. Overexpression of MIC19 Inhabits Collapsed Mitochondrial Membrane Potential and Oxidative Stress Induced by OxyHb In Vitro

**Figure 7:**
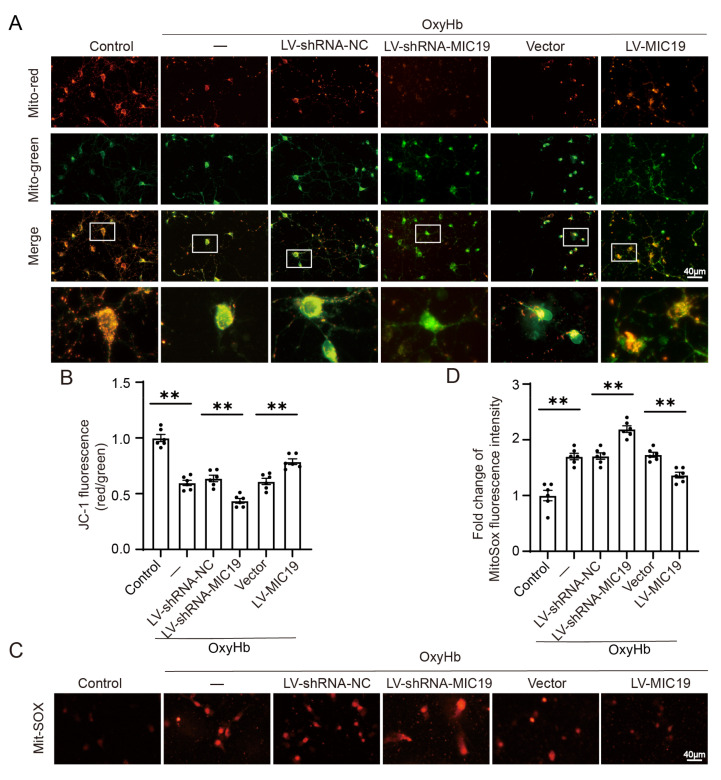
Effects of knockdown and overexpression of MIC19 on mitochondrial function in vitro. (**A**,**B**) JC-1 was applied to detect the MMP. Normal mitochondria presented the red fluorescence aggregated. However, red fluorescence converted into green if mitochondria suffered damage, whereas green fluorescence signified a decreased ΔΨm (** *p* < 0.01 vs. sham: *p* < 0.0001; ** *p* < 0.01, LV-shRNA-NC vs. LV-shRNA-MIC19: *p* < 0.0001; ** *p* < 0.01, vector vs. LV-MIC19: *p* = 0.0002; n = 6, ANOVA type: ordinary one-way ANOVA). Scale bar = 40 μm, magnification: 40× mirror. (**C**,**D**) Mitochondrial ROS production changed, followed by the MIC19 overexpression and knockdown in cultured neurons; the red fluorescence signified mitochondrial ROS activity (** *p* < 0.01 vs. sham: *p* < 0.0001; ** *p* < 0.01, LV-shRNA-NC vs. LV-shRNA-MIC19: *p* < 0.0001; ** *p* < 0.01, vector vs. LV-MIC19: *p* = 0.0007; n = 6, ANOVA type: ordinary one-way ANOVA). Scale bar = 40 μm, magnification: 40× mirror. Mean ± SE was used to describe all data, and mean values in the sham group were normalized to 1.0.

As to mitochondrial damage, JC-1 was applied to detect the mitochondrial membrane potential (MMP), aiming to explore the effects of MIC19 in mitochondria. Normal mitochondria presented the red fluorescence aggregated. However, red fluorescence was converted into green if mitochondria suffered damage. The results showed that red fluorescent intensity decreased and green fluorescent intensity increased after being treated with OxyHb. Furthermore, in the OxyHb + LV-MIC19 group, the effect was inverted compared with in the OxyHb group, and it means that MIC19 overexpression reduced the mitochondrial damage induced by ICH. However, in the OxyHb + LV-shRNA-MIC19 group, the green fluorescent intensity increased more than that in the OxyHb + LV-shRNA-NC group (Figure 7A,B). A MitoSOX Staining Kit was used to explore the effect of MIC19 in mitochondrial oxidative stress. The red fluorescent intensity increased after being treated with OxyHb. However, the effect was inverted in the OxyHb + LV-MIC19 group (Figure 7C,D).

### 2.9. MIC19 Exerts Its Protective Role via Maintaining the Stability of MIC19–SAM50 Axis

**Figure 8:**
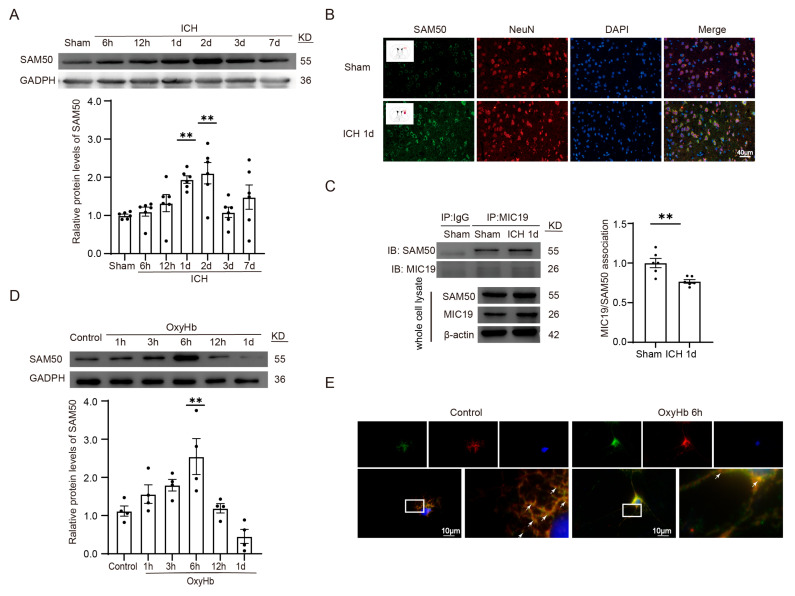
MIC19 participates in ICH-induced mitochondrial dysfunction by enhancing the stability of the MIC19–SAM50 axis. (**A**) The protein levels of SAM50 at 6 h, 12 h, 1 d, 2 d, 3 d, and 7 d after ICH treatment detected by Western blotting (** *p* < 0.01, 1 d: *p* = 0.0095 vs. sham group, n = 6, ANOVA type: ordinary one-way ANOVA). (**B**) Immunofluorescent analysis performed with SAM50 antibodies (green), neuronal marker NeuN (red), and then DAPI (blue) in coronal section nuclei. Scale bar = 40 μm, magnification: 40× mirror. (**C**) The MIC19/SAM50 interactions using CO-IP (** *p* < 0.01, 1 d: *p* = 0.0040 vs. sham group, n = 6). (**D**) Western blot was used to analyze the changes in SAM50 protein levels at 1 h, 3 h, 6 h, 12 h, and 1 d after being treated by OxyHb in vitro (** *p* < 0.01, 6 h: *p* = 0.0032 vs. sham group, n = 4, ANOVA type: ordinary one-way ANOVA). (**E**) Immunofluorescent analysis performed with MIC19 antibodies (green), SAM50 antibodies (red) and then DAPI (blue) in primary-cultured cortical neurons. Amounts of arrows displays the connection strength of the SAM50–MIC19 axis. Scale bar = 10 μm, magnification: 2× mirror. Mean ± SE was used to describe all data, and mean values in the sham group were normalized to 1.0.

Finally, we included some experiments to explore the mechanism of the protective role of MIC19 in ICH-induced SBI. In vivo, a significant increase in SAM50 protein levels was found at 24 h after ICH modeling (Figure 8A). Furthermore, we performed immunofluorescent staining to explore the cellular distribution and expression of SAM50 (Figure 8B). Then, CO-IP was utilized to explore the relationship of MIC19 with SAM50. According to the results, the binding of MIC19 to SAM50 was interrupted upon intracerebral hemorrhage, and the overexpression of MIC19 and SAM50 might compensate for the interaction strength between MIC19 and SAM50 to maintain the mitochondrial structure (Figure 8C). In vitro, a significant increase in SAM50 protein levels was found at 6 h after modeling via Western blot (Figure 8D). Finally, in the immunofluorescent experiments, we also found that the cellular distribution of the two proteins (MIC19 and SAM50) in primary-cultured cortical neurons coincided, which may further verify their relationship (Figure 8E).

## 3. Discussion

The role of mitochondria in preventing neuronal death and improving neurological function in intracerebral hemorrhage has been widely discussed. However, the molecular mechanisms behind this phenomenon remain unclear [23]. As a dynamic, interconnected network, mitochondria can perform different interconnected functions and also take part in the processes of autophagy, apoptosis, and other cellular stress responses [24]. Mitochondrial cristaes are dynamic, bioenergetic compartments, and the cristae junctions are closely related to the reaction of oxidative phosphorylation, the protein import in mitochondria, and apoptosis. Furthermore, the shape of mitochondria cristae changes with different physiological situations [25,26]. As a key component in mitochondria morphology, the MICOS complex has other biological functions such as energy generation [27]. However, the effects and exact mechanisms of the MICOS complex and its related components, especially MIC19 in ICH-induced SBI, are still unclear.

Junhui Tang et al. reported that MIC19 is an essential part of the MICOS complex and takes part in assembling the mitochondrial intermembrane space bridging (MIB) complex. Furthermore, MIC19 cleavage destroys the integrity of the MIB complex, causes the loss of mitochondrial cristae junctions, and leads to abnormality of the mitochondrial morphology [19,28]. In our study, MIC19 protein levels were found to be significantly upregulated after modeling, especially 24 h after ICH modeling in rats and at 6 h after being treated by OxyHb in cultured neurons. Additionally, MIC19 overexpression alleviated the subsequent injury after ICH by mitigating neuronal death and neurodegeneration, oxidative stress, neurological impairment, crista junction collapse, mitochondrial structure abnormality, and cognitive dysfunction. Conversely, MIC19 knockdown had the opposite effect. Therefore, it is concluded that MIC19 plays a protective role in ICH-induced SBI.

As a core component in the MICOS complex, MIC19 is an essential part in cristae patterning, MICOS complex assembling, and MIB complex stability. Its ablation affects the mitochondrial dynamics and causes the breakage of the MICOS complex. Further studies have confirmed that MIC19 can also take part in linking MIC60 or SAM50 to establish the SAM50–MIC19–MIC60 axis, and the axis connects the SAM and MICOS complexes to maintain the stability of mitochondrial cristae architecture and functions [17,19,28,29]. Furthermore, as Manjula Darshi reported, the loss of MIC19 can lead to abnormality of the mitochondrial morphology and impair the bioenergetic function of cells, such as glycolytic lactate production [30]. In our latest findings, the mitochondrial structure suffered damage, and more neurons faced death in the ICH group compared with in the sham group. However, MIC19 overexpression could reverse these bad effects. These results indicate that MIC19 overexpression plays an important role in SBI after ICH, including in avoiding mitochondrial structure damage and reducing cell death and apoptosis.

It was reported that MIC19 associated with complex IV and MIC19 knockdown led to the reduction of complex IV expression and oxidative phosphorylation, while no changes were found in complex III and V [20]. Junhui Tang also reported that MIC19 interacts with SAM50 to regulate crista junction formation, which plays an important role in mitochondrial energy production. They also found that the interruption of the SAM50–MIC19 axis destroys the mitochondrial structure and significantly reduces energy generation, even if the SAM and MICOS complexes still exist [19]. In our study, MIC19 overexpression increased Cytochrome c Oxidase activity, which may be advantageous to mitochondrial functions, such as ATP production, and energy metabolism. Furthermore, we found that the binding between MIC19 and SAM50 in the brain tissues was broken off after intracerebral hemorrhage. The overexpression of MIC19 and SAM50 may compensate for the damage induced by the weakening of the MIC19–SAM50 interaction. Thus, we hypothesize that the increase in MIC19 caused by ICH might maintain the stability of the SAM50–MIC19 axis to protect the brain tissues from the damage from ICH.

There were some limitations in our study. First of all, we did not randomize gender and age. Only adult male rats were included in the research. Second, our ICH model was made via injecting the autologous blood obtained from the caudal artery of rats. The method cannot fully mimic the occurrence of ICH owing to microvascular rupture in humans. Third, we only used OxyHb to treat the cultured neurons, mimicking ICH in vitro. However, blood from microvascular rupture also contains other components in addition to OxyHb, such as inflammatory factors, plasmin, platelets, etc. Fourth, it was actually difficult for us to differentiate the effect in rats induced by neurons or glial cells after ICH modeling in vivo. Finally, although the functions of MIC19 in ICH-induced SBI were evaluated, the interaction with other molecules in ICH and the upstream signaling pathways was not thoroughly investigated. For example, ICH occurrence can activate c-JUN N-terminal kinase (JNK), which is upstream of RB1-inducible coiled-coil 1 (RB1CC1), and RB1CC1 upregulation can stimulate the generation of MIC19 [31]. ICH can activate the protein kinase R-like endoplasmic reticulum kinase (PERK) that phosphorylates O-linked N-acetylglucosamine transferase (OGT). Phosphorylated OGT glycosylates TOM70 on Ser94, enhancing MIC19 protein import into mitochondria and promoting cristae formation [32,33]. These molecules might take part in the pathological process and need further exploration.

## 4. Methods and Materials

Table S1 summarizes the interventions and materials in our study.

### 4.1. Animals

All of animal experiments in the research obtained the authorization of the Animal Care Committee of the First Affiliated Hospital of Soochow University and were carried out according to the guidelines formulated by the National Institutes of Health (ethical approval reference number: SDFYY-2019278). All adult male Sprague Dawley (SD) rats (body weight: 250–300 g, RRID: MGI:5651135) were provided by the Chinese Academy of Sciences in Shanghai, China. In our study, we made efforts to provide comfortable feelings for the experimental rats by applying, for example, suitable room temperature (24 °C), adequate food and water, spacious room, and a 12 h light/dark cycle. Furthermore, our animal data reporting strictly followed the Animal Research: Reporting of In Vivo Experiments (ARRIVE) 2.0 guidelines [34]. Each rat was fixed with an earmark and randomly allocated to the pre-designed groups via a created random number in Microsoft Excel 2019 (RAND). Table S2 shows the specific quantity of rats each experiment used.

During the experiments, we exerted effort to take steps to minimize the suffering of rats such as heating pad preparation to keep them warm and adequate anesthesia to alleviate pain, and the experiments were operated by skilled researchers.

### 4.2. Experimental Design

In Experiment 1, all sequentially numbered rats were randomly grouped into the interventional groups by a technician not involved in this experiment. As shown in Table S2, 42 rats were finally allocated to the pre-designed seven groups (n = 6 per group) after modeling in Experiment 1: one sham group and six ICH groups, namely 6 h, 12 h, 1 d, 2 d, 3 d, and 7 d after ICH groups, which were divided according to the time points after ICH modeling. All grouped rats were anesthetized by isoflurane gas before operation. After ICH modeling, their cerebral tissues were perfused with phosphate-buffered saline (PBS) transcranially, taken out by skilled researchers in 1 min, and collected for subsequent analysis (Western blot and immunofluorescent analysis).

In Experiment 2, rats were randomly assigned using the same method described above and were divided into six groups according to the interventions: sham group, ICH group, ICH + LV-shRNA-NC group, ICH + LV-shRNA-MIC19 group, ICH + vector group, ICH + LV-MIC19 group (22 rats per group). MIC19 was knocked down and overexpressed by administering the related lentiviral vector to the lateral ventricles of rats. Next, all grouped rats were used for ICH modeling 7 days after the virus administration. Their cerebral tissues were perfused with PBS, taken out by skilled researchers in 1 min, and collected for subsequent analysis one day after ICH modeling. Six rats in the pre-designed six groups were used for Western blot analysis, TUNEL, and fluoro-jade B (FJB) staining. The other ten rats in the pre-designed six groups were used to perform the behavioral tests, including adhesive removal tests, the Morris water maze, and rotarod tests. Another six rats in the pre-designed six groups were used for malondialdehyde (MDA) and superoxide dismutase (SOD) detection. The last six rats in the pre-designed six groups were utilized for electron microscopy to evaluate the microstructure of the mitochondria. Two observers who were blinded to the groupings took part in counting and statistically analyzing these mentioned tests.

In vitro, all grouped cultured neurons were exposed to OxyHb to mimic ICH 7 days after the virus administration. Then, the samples were collected for subsequent analysis six hours after ICH. We finally researched the differences among these six groups, which were divided according to the interventions (sham group, OxyHb group, OxyHb + LV-shRNA-NC group, OxyHb + LV-shRNA-MIC19 group, OxyHb + vector group, OxyHb + LV-MIC19 group) for analysis of protein expression, neuronal death, mitochondrial functions, and oxidative stress via Western blot analysis, live–dead cell staining, mitochondrial membrane potential detection, and MitoSOX measurement.

In Experiment 3, we also performed Western blot analysis, co-immunoprecipitation, and immunofluorescence analysis to explore the potential mechanism of MIC19 in ICH-induced SBI.

### 4.3. Rat Model of ICH

We utilized an autologous blood model because this model can closely simulate clinical ICH [22,35,36]. During the establishment of the ICH model, the vital signs of each rat were monitored by the researchers, and their rectal temperature was kept at 36.5–37.5 °C. Briefly, rats were anesthetized (isoflurane gas anesthesia) and fastened via a stereotaxic apparatus. Next, the scalp was incised to expose the skull and then a hole was drilled in the skull via a driller above the right basal ganglia of every rat (3.5 mm to the right of the anterior fontanelle and then 0.2 mm posterior to the bregma). A microliter syringe was fixed in the apparatus and slowly inserted through the hole (5.5 mm in depth). Then, 100 μL of autologous blood obtained from the caudal artery of rats was slowly injected into the well-confirmed location at a speed of 20 μL/min. After injection, the microliter syringe was prohibited from being removed immediately; instead, the researcher was required to wait 5 min before slowly withdrawing it. In the sham group, 100 μL of physiological saline was injected intracerebrally as a control. Finally, the hole was blocked with bone wax, and the scalp incision was sewed up.

### 4.4. Primary-Cultured Cortical Neurons and ICH Induction

Based on our previous literature, primary-cultured cortical neurons were isolated from the cortical tissues of 18-gestational-days-old fetal rats [22,36]. First, these cortical tissues of the fetal rats were collected, and meninges or vessels were removed. Next, the obtained samples were digested with 0.25% trypsin (ThermoFisher, Waltham, MA, USA) for 5 min at 37 °C, and the digested samples were washed with PBS. Then, the digested samples were blown gently and filtered to acquire the brain suspension. The sediments were kept, and the supernatants were trashed after centrifuging the suspension for 5 min at 1000× *g*. The sediment was then resuspended via the medium (neurobasal medium containing: 2% B27, 50 U/mL of streptomycin, 50 U/mL of penicillin, and 0.5 mM of GlutaMAX, all from Gibco). Finally, plates with six wells, twelve wells, or twenty-four plates were used to seed and culture the isolated cells in the neurobasal medium and were placed in humidified conditions at a constant temperature of 37 °C and 5% CO_2_. After the procedures described above, half of the neurobasal medium in the plates needed to be changed every two days for one week. As for ICH induction, each well was treated using 5 μM of OxyHb at 37 °C to imitate ICH in neurons for further research.

### 4.5. Intracerebral Lentivirus Injections

To downregulate and upregulate the level of MIC19, four virus agents of LV-MIC19, vector, LV-shRNA-MIC19, and LV-shRNA-NC were injected in the lateral ventricle through the borehole using a stereotaxic instrument 7 days before ICH modeling. We drilled a hole on the left of the skull above the lateral ventricles of the rats which was located 1.1 mm posterior and 1.5 mm lateral to the bregma and slowly injected the lentivirus with a speed of 0.5 μL/min into the lateral ventricle, which was 3.5 mm in depth. Finally, we waited five minutes and then slowly withdrew the syringe after injection to avoid the regurgitation of the liquid and sealed the hole with bone wax. In the lentiviral vector transductions, a marker protein GFP was not encoded in order to not affect the use of fluorescent channels.

LV-shRNA-MIC19 sequences: Vector: GV112 (Vector information: General: 7402 bp; HIV-1_5_LTR, truncHIV-1_3_LTR: 844–1024; psi: 1076–1213; RRE: 1695–1928; ORF frame 1: 1573–2460; hU6 Promoter: 2632–2899; Polylinker: 2900–2929; CAG_enhancer: 3040–3327; CMV promoter: 2976–3564; Purmycin: 3588–4187; loxP: 4202–4235; WPRE: 4293–4880; ORF frame 2: 4394–5434; HIV-1_5_LTR, truncHIV-1_3_LTR: 5408–5588; pBR322_origin: 6254–5635; Ampicillin: 7269–6409; AmpR_promoter: 7339–7311; CAG_enhancer: 327–614; CMV_immearly_promoter: 248–819. Primer locations and sequences: pGCSIL-F(2667–2687): CCATGATTCCTTCATATTTGC).

1. Sense: 5′-GTCCTCTCCATCTGGCTCTAA-3′. titer: 4.5 × 10^8^ TU/mL;
2. Sense: 5′-AACCGCACAAAGGTGAAGCAT-3′. titer: 4.5 × 10^8^ TU/mL;
3. Sense: 5′-GAGGAGCTGGCATTGGAACAA-3′. titer: 3 × 10^8^ TU/mL (last choice).

LV-shRNA-NC sequences: Vector: GV341 (Vector information: General: 10,393 bp; HIV-1_5_LTR, trunkHIV-1_3_LTR: 835–1015; psi: 1067–1204; RRE: 1680–1913; ORF frame 1: 1558–2445; Ubiquitin Promoter: 2617–3833; 3FALG: 3910–3984; SV40 promoter: 3991–4368; Puromycin: 4376–4975; WPRE: 5001–5588; HIV-1_5_LTR, truncHIV-1_3_LTR: 6112–6292; pBR322_origin: 9159–8540; Ampicillin: 10,174–9314; AmpR_promoter: 10,244–10,216; CAG_enhancer: 318–605; CMV_immearly_promoter: 239–810. Primer locations and sequences: Ubi-F(3756–3778): GGGTCAATATGTAATTTTCAGTG).

Sense: 5′-TTCTCCGAACGTGTCACGT-3′. titer: 2.5 × 10^8^ TU/mL.

### 4.6. Western Blot

Based on our previous study [37], the brain tissues were stored at −80 °C and then the brain samples were ground and lysed with lysis buffer and phenylmethylsulfonyl fluoride (PMSF) so that we could obtain suspensions of the brain tissues of each rat. The supernatants were kept, and the sediments were trashed after centrifuging the suspension for 10 min at 12,000× *g* at 4 °C. Then, the protein concentrations were detected via bicinchoninic acid (BCA) protein assays (BioSwamp, Wuhan, China). Protein samples in each group (20 μg/lane) and a suitable marker (BioSwamp, Wuhan, China) were added into the 12% well-prepared SDS–PAGE, and the proteins were separated owing to the protein molecular weight and then transferred to a NC membrane. After being blocked in 5% non-fat milk at room temperature for 1 h, the membrane was then soaked in well-prepared primary antibodies (MIC19 (Abcam, Waltham, MA, USA)) at 4 °C for at least 8 h. After being washed with PBST (PBS + 0.1% Tween), the membrane was again soaked in the well-prepared secondary antibody (Affinity, Shanghai, China) for 1 h and then washed again with PBST. Finally, blot detection was performed via a chemiluminescence kit (Millipore, Burlington, MA, USA) and the visualization system (Bio-Rad, Hercules, CA, USA) and then the bands of target proteins were quantified via the software ImageJ V1.8.0 (NIH, Stapleton, NY, USA).

### 4.7. Immunofluorescent Microscopy

Based on our previous reports [35], immunofluorescent staining is a good method to observe the expression of a protein in cortical cells around a hematoma. Brain samples were collected, embedded in paraffin wax, and then cut to 4 μm thickness for further research. After dewaxing, antigen retrieval, and non-specific binding blocking, the 4-micrometer-thick brain slices were soaked in well-prepared primary antibodies (MIC19 and NeuN) at 4 °C for at least 8 h. Subsequently, after soaking in the well-prepared secondary antibodies at 37 °C for 1 h in the dark, the brain slices in the slide were coverslipped with 4′-6-diamidino-2-phenylindole (DAPI). In the procedures, it was critical for us to keep the cover slide clean and avoid bubbles being generating. Finally, a fluorescent microscope (Nikon, Minato City, Japan) was used for observation after the slide dried, and ImageJ software V1.8.0 was used for quantifying.

### 4.8. TUNEL and FJB Staining

TUNEL staining was used to measure cell apoptosis. First, the brain samples were embedded in paraffin wax and cut to 4 μm thickness. After being dehydrated under 70 °C conditions, the wax covering the brain slices was dewaxed with xylene and ethanol with different concentrations. After these procedures, the brain slices were then soaked in TUNEL reagent (Beyotime, Shanghai, China) at 37 °C for 1 h and then in NeuN at 4 °C for at least 8 h. Subsequently, after soaking in well-prepared secondary antibodies at 37 °C for 1 h in the dark, the brain slices in the slide were covered with DAPI. Finally, an observer who was blinded to the groupings took part in counting and statistically analyzing TUNEL-positive cells via a fluorescent microscope (Nikon, Japan). The percentage of TUNEL-positive cells was used to assess the neuronal death ICH induced.

According to the guidelines of previous studies [38,39], FJB staining can be utilized to show the death of neurons in rats’ cortex and the perihematomal region. The brain sections were soaked in sequence in 80% alcohol + 1% NaOH (5 min), 70% ethanol (2 min), 0.06% potassium permanganate (10 min), and 0.0004% FJB working solution (30 min). After being dried at 55 °C for 10 min and cleared with xylene, the brain sections were finally coverslipped with neutral resin. A fluorescent microscope (Nikon, Japan) was used to visualize FJB-positive neurons. An observer who was blinded to the groupings took part in counting and statistically analyzing FJB-positive cells.

### 4.9. Electron Microscope

Based on the above statements, six interventional groups (sham group, ICH group, ICH + LV-shRNA-NC group, ICH + LV-shRNA-MIC19 group, ICH + vector group, ICH + LV-MIC19 group) were utilized for the electron microscopy. The brain tissues were taken out in 1 min after the death of rats and put in glutaraldehyde fixed solution immediately. Then, we picked out 3–5 small particles with a toothpick and put them into a 1.5 mL pointed Eppendorf (EP) tube which was prepared with the glutaraldehyde fixed solution in advance. Finally, we stored them at 4 °C and sent them to Biomisp (Wuhan, Hubei, China) for electron microscopy.

### 4.10. Mitochondrial Morphology Analysis

To research the mitochondrial morphology under different interventions, the mitochondrial morphology was visually divided into five classifications (tubular, short tubular, fragmented, expanded, and large spherical) and quantified according to the reports of the related literature [19]. An observer who was blinded to the groupings took part in counting and statistically analyzing the change in the percentage of each mitochondrial morphology in the six interventional groups. Crista junctions are also an important microstructure for mitochondria, and an observer who was blinded to the groupings was also arranged to calculate the percentage of crista junctions in the total cristae.

### 4.11. Neurological Scoring

At 1 d after ICH modeling, behavioral impairments of injured rats were tested through a publicly available scoring system containing seven individual tests (Table 1) [22]. One observer who was blinded to the groupings performed all behavioral testing, and the results were recorded by another person blinded to the experiment.

### 4.12. Adhesive Removal Test

Based on previous guidelines [22,40], the adhesive removal test was implemented for measuring the motor coordination and sensation of rats. A 9 mm sticker was affixed to the palm of each forepaw of the rats, and the length of time it took for rats to get rid of the stickers was recorded. We spent 3 days training the rats before ICH modeling to make sure that each rat in different experimental groups had the ability to remove the sticker, and we recorded the time for the rats to remove the stickers in these three days as the baseline data. Then, this test was repeatedly implemented at the following time points: 1 d, 5 d, 7 d, 10 d, 14 d, 21 d, and 28 d after ICH modeling. One observer who was blinded to the groupings performed the test, and the results were recorded by another person blinded to the experiment.

### 4.13. Rotarod Test

As described in previous studies [22,41], locomotor impairments of rats were assessed via the rotarod test. Each rat was placed on the 10 cm diameter rotarod cylinder, and the time that the rats stayed on the cylinder was recorded. The speed of the cylinder increased from 4 to 30 revolutions per minute constantly within 60 s and was sustained for 300 s. If rats dropped or gripped the device, the experiment ended, and the stopping time was recorded. Before ICH modeling, we spent 3 days training the rats to make them have the ability to move on the rod, and we recorded the time in the three days as the baseline level. This test was performed at the following time points: 1 d, 5 d, 7 d, 10 d, 14 d, 21 d, and 28 d after ICH modeling. One observer who was blinded to the groupings performed the test, and the results were recorded by another person blinded to the experiment.

### 4.14. Morris Water Maze

According to the previous report [22,42,43], the cognitive ability of the rats after ICH modeling was assessed via the Morris water maze. A 180 cm diameter round pool and a 10 cm diameter high platform were placed in a fixed position to form the experimental scene. Each rat was placed in the quadrant opposite to the quadrant in which the platform was located, which allowed it to arrive at the underwater platform within 60 s. Finally, we kept every rat on the platform for 15 s in order to strengthen their memory of the location of the platform. One observer who was blinded to the groupings performed the test, and the results were recorded by another person blinded to the experiment.

### 4.15. Live–Dead Cellular Staining

Cell apoptosis was assessed via a calcein acetoxymethyl (AM)/propidium iodide (PI) double-staining kit. After being washed with PBS, the neurons were soaked in a well-prepared reagent containing calcein AM and PI for 30 min at 37 °C in the dark. Finally, the cortical neurons in a 24-well plate on the cover glass were coverslipped on the slides, and a fluorescent microscope was used for evaluating the apoptotic ratio (Nikon, Japan).

### 4.16. MitoSOX Staining

The MitoSOX Red (Thermo Fisher Scientific, Waltham, MA, USA) assay can be used to measure mitochondrial ROS activity, as previous studies reported [44]. After treating primary-cultured cortical neurons, which were transfected by a virus, with OxyHb, we added 13 μL of DMSO in a vial of MitoSOX Red indicator containing 50 μg of content to make 5 mM MitoSOX. Then, PBS was used to dilute 5 mM MitoSOX to prepare the 5 μM MitoSOX reagent working solution. Furthermore, the cortical neurons on the coverslip in a 24-well plate were soaked in the well-prepared 5 μM MitoSOX reagent working solution at 37 °C for 10 min in the dark and then washed three times using PBS. Finally, we detected the ROS fluorescent intensity using a fluorescent microscope (Nikon, Japan).

### 4.17. Malondialdehyde (MDA) and Superoxide Dismutase (SOD) Detection

A malondialdehyde (MDA) kit (Abcam) and superoxide dismutase (SOD) kit (Nanjing Jiancheng Bioengineering Institute, Nanjing, China) were used to detect the signs of oxidative stress. The experimental procedures were implemented strictly according to the manufacturer’s instructions.

### 4.18. Mitochondrial Membrane Potential Detection

JC-1 staining was used for detecting MMP on the basis of the manufacturer’s instructions (Beyotime, China). After the OxyHb and virus interventions, PBS was used to wash the neurons and then the neurons were soaked in the well-prepared JC-1 staining working solution (20 min, 37 °C). Finally, an observer who was blinded to the groupings took part in counting and statistically analyzing the stained cells.

### 4.19. Cytochrome c Oxidase Activity Colorimetric Assay

The Cytochrome c Oxidase Activity Colorimetric Assay Kit (Biovision, Milpitas, CA, USA) was used to detect the activity of respiratory complex IV, a core component in mitochondria for energy production. On the basis of the manufacturer’s instructions (Biovision, Milpitas, CA, USA), 5–10 μL fresh test samples were added to each well of a 96-well plate for measuring the activity of Cytochrome c Oxidase. As for the negative control samples, we used an equal amount of dilution buffer. Then, 120 μL of diluted cytochrome c (reduced cytochrome c: 20 μL, cytochrome assay buffer: 100 μL) was added to each sample, then shaken, and the reduction in optical density (OD) was immediately recorded over 30–45 min. Finally, the reaction rate was calculated (Cytochrome Oxidase Activity (units/mg):(ΔOD/Time (Δt))/Ɛ protein (mg)).

### 4.20. Co-Immunoprecipitation Analysis

As illustrated previously [45], the preparation of samples was similar to that for Western blotting. First of all, the brain tissue was ground, lysed, centrifuged, and quantified for IP and Western blotting. Next, immunoprecipitation magnetic beads were cleaned with PBS and mixed with a well-prepared sample and MIC19 antibody (Thermofisher) or normal Rabbit IgG (negative control) (purchased in November 2020, Cell Signaling Technology) to form a protein–antibody–bead mixture and were agitated with a rotary agitator at 4 °C for at least 8 h. Then, cell lysis buffer was used to wash the mixtures and then the mixtures were denatured with 2× SDS loading buffer. In the final step, the relationship between SAM50 and MIC19 was detected via Western blotting.

### 4.21. Statistical Analysis

GraphPad Prism 9.0 (La Jolla, CA, USA) was used to analyze the data in our study. The data were displayed as the mean and standard error (SE) or the mean and standard deviation (SD). The Shapiro–Wilk test and Kolmogorov–Smirnov tests were used to make sure the data were normally distributed. Ordinary one-way ANOVA and two-way ANOVA were used to analyze differences between multiple groups, while the Tukey or Šídák’s test was used to analyze differences between two groups. A *p* < 0.05 was also defined as a significant difference. The statistics reporting is shown in Table S3.

## 5. Conclusions

**Figure 9:**
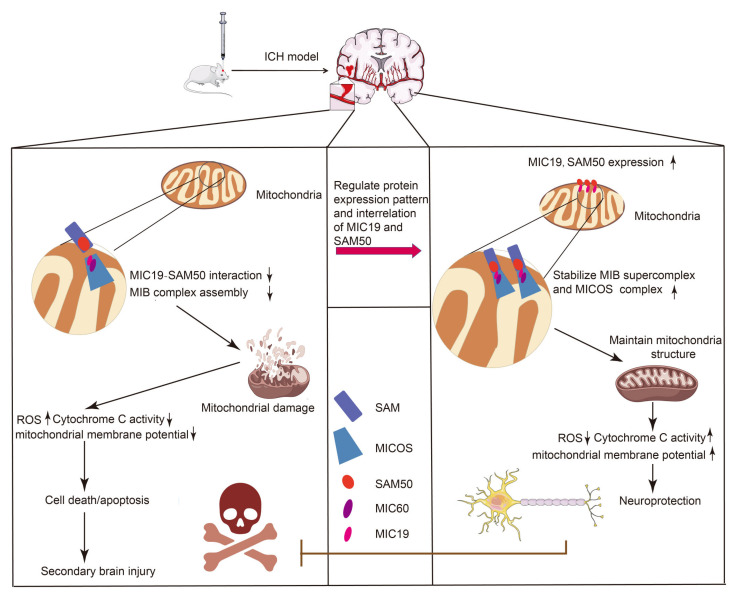
Diagram showing the effects of MIC19 after ICH. In ICH-induced SBI, MIC19 upregulation stabilized the MIC19–SAM50 axis and MIB super complex to maintain the stability of neuronal mitochondrial structure, which was disrupted by ICH, to alleviate neuronal damage. Then, a series of brain-protective measures was initiated, such as the reduction of ROS production in mitochondria, the increase in activity of Cytochrome c Oxidase, and the recovery of collapsed mitochondrial membrane potential. Short arrows upward means increase and short arrows downward means decrease.

In summary, our research demonstrated that upregulation of MIC19 expression can mitigate neuronal degeneration and death, ameliorate behavioral impairments and cognitive dysfunction, and stabilize mitochondrial dysfunction. Specifically, the increase in MIC19 levels confers a protective effect by enhancing the stability of the MIC19–SAM50 axis during ICH (Figure 9). Our findings suggest that MIC19 plays a protective role in ICH-induced SBI and could be a novel therapeutic target in ICH treatment.

## Figures and Tables

**Table 1:** Neuroscore scoring criteria for the sub-tests.

Sub-Test	0	1	2	3
Spontaneous activity (SA)	Animal was akinetic	Animal moved slowly or minimally	Animal approached 1–2 walls	Animal approached at least 3 walls of the cage or raised on hindlimbs to explore the top of the cage
Body proprioception (BP)		Animal had a unilateral response	Animal had either a weak bilateral response or weak left response and brisk right response	Animal had a brisk bilateral response
Vibrissae touch (VT)		Animal had a unilateral response	Animal had either a weak bilateral response or weak left response and brisk right response	Animal had a brisk bilateral response
Limb symmetry (LS)	Hemiparesis	Left forelimb or left hindlimb fixed	Asymmetric extension	All limbs were extended symmetrically
Lateral turning (LT)	Animal had no turning at all on one side	Animal had unequal turning	Animal turned bilaterally less than 45° on both sides	Animal turned bilaterally at least 45° on both sides
Forelimb outstretching (FO)	Animal had a paretic forelimb	Animal walked in circles	Animal walked asymmetrically or to one side	Animal briskly walked symmetrically on forepaws
Climbing (CL)		Animal failed to climb or circled instead of climbing	Animal climbed to the top and had a weak grip or animal climbed but had a strong grip	Animal climbed to the top and had a strong grip

## Data Availability

The data that support the results of this study are available from the corresponding author of the article upon reasonable request.

## Supplementary Materials

The supporting information can be downloaded at: https://www.mdpi.com/article/10.3390/ijms241411553/s1.

## Abbreviations

ARRIVE: Animal Research: Reporting of In Vivo Experiments; BBB: blood–brain barrier; BCA: bicinchoninic acid; CJs: cristae junctions; MIB: mitochondrial intermembrane space bridging; ICH: intracerebral hemorrhage; MICOS: mitochondrial contact site and cristae organization system; OxyHb: oxygen hemoglobin; OD: optical density; PBI: primary brain injury; PMSF: phenylmethylsulfonyl fluoride; PBS: phosphate-buffered saline; SBI: secondary brain injury; SD: Sprague Dawley; ROS: reactive oxygen species.
