# Supplementary material for: MIC19 Exerts Neuroprotective Role via Maintaining the Mitochondrial Structure in a Rat Model of Intracerebral Hemorrhage
Source: Int J Mol Sci. 2023 Jul 17;24(14):11553. doi: 10.3390/ijms241411553 (PMC10380515; doi:10.3390/ijms241411553)
Supplement: Supplementary file 1 [file ijms-24-11553-s001.zip › ijms-2473532-supplementary.pdf]

MIC19 Exerts Neuroprotective Role Via Maintaining the Mitochondrial Structure in a Rat Model of Intracerebral Hemorrhage

Siyuan Yang [1,2,†], Xulong Yin [2,3,†], Jiahe Wang [1,2], Haiying Li [1,2], Haitao Shen [1,2], Qing Sun [1,2,*] and Xiang Li [1,2,*]

[1] Department of Neurosurgery & Brain and Nerve Research Laboratory, The First Affiliated Hospital of Soochow University, Suzhou 215006, China
[2] Institute of Stroke Research, Soochow University, Suzhou 215006, China
[3] Department of Neurology, The First Affiliated Hospital of Soochow University, Suzhou 215006, China
* Correspondence: qsun@suda.edu.cn (Q.S.); xiangli2017@suda.edu.cn (X.L.)
† These authors contributed equally to this work.

Table S1 List of materials

Methods	Reagents	Source	Research Resource Identifiers & catalogue numbers
	Sprague Dawley rats	Experimental Animal Center of the Chinese Academy of Sciences	RRID:MGI:5651135
WB	MIC19	Abcam	Cat#ab224565
	Sam50	Santa Cruz Biotechnology	Cat#sc-100493
	GADPH	Affinity Biosciences	Cat#AF7021 RRID:AB_2839421
	β-Tubulin	Cell Signaling Technology	Cat#2128
IF	MIC19	Abcam	Cat#ab224565
	Sam50	Santa Cruz Biotechnology	Cat#sc-100493
	NeuN	Abcam	Cat#ab104224 RRID:AB_10711040
	DAPI	Thermo Fisher Scientific	Cat#D1306, RRID:AB_2629482
	Alexa Fluor-555	Invitrogen	Cat#A31570, RRID:AB_2536180
	Alexa Fluor-488	Invitrogen	Cat#A21206, RRID:AB_2535792
COIP	MIC19	Thermo Fisher Scientific	Cat#A305-497A
	Sam50	Santa Cruz Biotechnology	Cat#sc-100493
	anti-rabbit IgG-HRP	Santa Cruz Biotechnology	Cat#sc-2004, RRID:AB_631746
	anti-mouse IgG-HRP	Santa Cruz Biotechnology	Cat#sc-2005, RRID:AB_631736
	anti-rabbit IgG Specific Light Chain	Abbkine Scientific Co, Ltd	Cat#ABM40168
	anti-mouse IgG Specific Light Chain	Abbkine Scientific Co, Ltd	Cat#A25012, RRID:AB 2737290
Other interventions	Tunel	Beyotime	Cat#C1091
	FJB	Biosensis	Cat#TR-150-FJB
	Live-dead cell staining	Thermo Fisher Scientific	Cat# L13152
	JC-1	Beyotime	Cat#C2008S
	Mit-SOX	Thermo Fisher Scientific	Cat#M36008
	Cytochrome C oxidase	Biovision	Cat#K287
	MDA	Beyotime	S0131S
	SOD	Beyotime	S0109

Table S2

In vivo test

Experiment 1

Group	total	dead	survived	Time of death	Survival rate
Sham	6	0	6	N/A	100%
ICH 6 h	7	1	6	After surgery for 12h	86%
ICH 12 h	8	2	6	1 during surgery and 1 during anesthesia	75%
ICH 24 h	8	2	6	During surgery	75%
ICH 48 h	7	1	6	After surgery for 24 h	86%
ICH 72 h	7	1	6	During anesthesia	86%
ICH 168h	8	2	6	After surgery for 24h	75%

Experiment 2

Group	total	dead	survived	Time of death	Survival rate
Sham	28	0	28	N/A	100%
ICH	30	2	28	2 during surgery	93.3%
ICH+LV-shRNA-NC	31	3	28	2 during surgery and 1 after surgery for 24 h	90.32%
ICH+LV-shRNA-MIC19	32	4	28	2 during surgery and 1 during anesthesia	87.5%
ICH+Vector	30	2	28	After surgery for 24 h	93.3%
ICH+LV-MIC19	31	3	28	After surgery for 24 h	90.32%

Experiment 3

Group	total	dead	survived	Time of death	Survival rate
Sham	7	1	6	1 during anesthesia	86%
ICH 6 h	8	2	6	1 during surgery and 1 during anesthesia	75%
ICH 12 h	7	1	6	1 during surgery	86%
ICH 24 h	8	2	6	1 during surgery and 1 after ICH for 12 h	75%
ICH 48 h	7	1	6	1 during surgery	86%
ICH 72 h	7	1	6	1 after ICH for 24 h	86%
ICH 168h	6	0	6	N/A	100%

Table S3 Statistics reporting

Figure	n/group	In vivo or in vitro	Test used	Statistic	P value	Stat-value	post-hoc test
1B	6	In vivo	Ordinary One-way ANOVA	F (6, 35) = 5.539	P=0.0004	*p< 0.05, 12h : p = 0.033, **p< 0.01, 1d: p = 0.0002, **p< 0.01, 2d: p = 0.0005, vs. Sham group	Šídák's multiple comparisons test
2A	6	In vivo	Ordinary One-way ANOVA	F (5, 30) = 12.64	P<0.0001	**p< 0.01 ICH vs. Sham: p = 0.0056; **p< 0.01, LV-shRNA-NC vs. LV-shRNA-MIC19: p = 0.0078; **p< 0.01, Vector vs. LV-MIC19: p = 0.0019	Šídák's multiple comparisons test
2C	6	In vivo	Ordinary One-way ANOVA	F (5, 30) = 20.1	P<0.0001	**p< 0.01, ICH vs. Sham: p <0.0001; **p< 0.01, LV-shRNA-NC vs. LV-shRNA-MIC19: p = 0.0058; **p< 0.01, Vector vs. LV-MIC19: p = 0.0047	Šídák's multiple comparisons test
2E	6	In vivo	Ordinary One-way ANOVA	F (5, 30) = 49.49	P<0.0001	**p< 0.01, ICH vs. Sham: p < 0.0001; **p< 0.01, LV-shRNA-NC vs. LV-shRNA-MIC19: p = 0.0008; **p< 0.01, Vector vs. LV-MIC19: p = 0.0002	Šídák's multiple comparisons test
2F	6	In vivo	Ordinary One-way ANOVA	F (5, 30) = 45.21	P<0.0001	**p< 0.01, ICH vs. Sham: p < 0.0001; **p< 0.01, LV-shRNA-NC vs. LV-shRNA-MIC19: p = 0.0002; **p< 0.01, Vector vs. LV-MIC19: p = 0.0016	Šídák's multiple comparisons test
3B	6	In vivo	Two-way ANOVA	F (20, 150) = 85.26	P<0.0001	Porportion of tubular crista: **p< 0.01, ICH vs. Sham: p < 0.0001; **p< 0.01, LV-shRNA-NC vs. LV-shRNA-MIC19: p < 0.0001; **p< 0.01, Vector vs. LV-MIC19: p = 0.0004	Tukey's multiple comparisons test
3C	6	In vivo	Ordinary One-way ANOVA	F (5, 30) = 32.85	P<0.0001	**p< 0.01, ICH vs. Sham: p < 0.0001; **p< 0.01, LV-shRNA-NC vs. LV-shRNA-MIC19: p < 0.0001;	Šídák's multiple comparisons test

						*p< 0.05, Vector vs. LV-MIC19: p = 0.0465	
4B	6	In vivo	Ordinary One-way ANOVA	F (5, 30) = 129.9	P<0.0001	**p< 0.01, ICH vs. Sham: p = 0.0005;<br>**p< 0.01, LV-shRNA-NC vs. LV-shRNA-MIC19: p = 0.0094;<br>*p< 0.05, Vector vs. LV-MIC19: p = 0.0139	Šídák's multiple comparisons test
4C	10	In vivo	Two-way ANOVA	F (5, 432) = 849.5	P<0.0001	**p< 0.01, ICH vs. Sham: p < 0.0001;<br>*p< 0.05, LV-shRNA-NC vs. LV-shRNA-MIC19: p = 0.0342;<br>**p< 0.01, Vector vs. LV-MIC19: p < 0.0001	Tukey's multiple comparisons test
4D	10	In vivo	Two-way ANOVA	F (5, 432) = 272.4	P<0.0001	**p< 0.01, ICH vs. Sham: p < 0.0001;<br>**p< 0.01, LV-shRNA-NC vs. LV-shRNA-MIC19: p = 0.0017;<br>**p< 0.01, Vector vs. LV-MIC19: p = 0.0010	Tukey's multiple comparisons test
4G	10	In vivo	Two-way ANOVA	F (5, 162) = 31.80	P<0.0001	**p< 0.01, ICH vs. Sham: p < 0.0001;<br>**p< 0.01, LV-shRNA-NC vs. LV-shRNA-MIC19: p < 0.0001;<br>**p< 0.01, Vector vs. LV-MIC19: p = 0.001	Tukey's multiple comparisons test
4H	10	In vivo	Two-way ANOVA	F (5, 162) = 24.26	P<0.0001	**p< 0.01, ICH vs. Sham: p < 0.0001;<br>**p< 0.01, LV-shRNA-NC vs. LV-shRNA-MIC19: p < 0.0001;<br>**p< 0.01, Vector vs. LV-MIC19: p = 0.0001	Tukey's multiple comparisons test
5A	6	In vivo	Ordinary One-way ANOVA	F (5, 30) = 37.56	P<0.0001	**p< 0.01, ICH vs. Sham: p < 0.0001;<br>**p< 0.01, LV-shRNA-NC vs. LV-shRNA-MIC19: p = 0.0002;<br>**p< 0.01, Vector vs. LV-MIC19: p = 0.0001	Šídák's multiple comparisons test
5B	6	In vivo	Ordinary One-way ANOVA	F (5, 30) = 24.51	P<0.0001	**p< 0.01, ICH vs. Sham: p < 0.0001;<br>**p< 0.01, LV-shRNA-NC vs. LV-shRNA-MIC19: p = 0.0002;<br>**p< 0.01, Vector vs. LV-MIC19: p = 0.0052	Šídák's multiple comparisons test

5C	6	In vivo	Ordinary One-way ANOVA	F (5, 30) = 23.77	P<0.0001	**p< 0.01, ICH vs. Sham: p < 0.0001;<br>**p< 0.01, LV-shRNA-NC vs. LV-shRNA-MIC19: p = 0.0009;<br>**p< 0.01, Vector vs. LV-MIC19: p = 0.0016	Šídák's multiple comparisons test
6A	4	In vitro	Ordinary One-way ANOVA	F (5, 18) = 3.814	P=0.0157	**p< 0.01, OxyHb 6h: p = 0.0096 vs. Control group	Šídák's multiple comparisons test
6B	4	In vitro	Ordinary One-way ANOVA	F (5, 18) = 9.950	P=0.0001	*p< 0.05, OxyHb vs. Control: p = 0.0168;<br>**p< 0.01, LV-shRNA-NC vs. LV-shRNA-MIC19: p = 0.0037;<br>**p< 0.01, Vector vs. LV-MIC19: p = 0.0039	Šídák's multiple comparisons test
6C	6	In vitro	Ordinary One-way ANOVA	F (5, 30) = 58.78	P<0.0001	**p< 0.01, OxyHb vs. Control: p < 0.0001;<br>**p< 0.01, LV-shRNA-NC vs. LV-shRNA-MIC19: p < 0.0001;<br>**p< 0.01, Vector vs. LV-MIC19: p = 0.0028	Šídák's multiple comparisons test
7B	6	In vitro	Ordinary One-way ANOVA	F (5, 30) = 51.75	P<0.0001	**p< 0.01, OxyHb vs. Control: p < 0.0001;<br>**p< 0.01, LV-shRNA-NC vs. LV-shRNA-MIC19: p < 0.0001;<br>**p< 0.01, Vector vs. LV-MIC19: p = 0.0002	Šídák's multiple comparisons test
7D	6	In vitro	Ordinary One-way ANOVA	F (5, 30) = 41.22	P<0.0001	**p< 0.01, OxyHb vs. Control: p < 0.0001;<br>**p< 0.01, LV-shRNA-NC vs. LV-shRNA-MIC19: p < 0.0001;<br>**p< 0.01, Vector vs. LV-MIC19: p = 0.0007	Šídák's multiple comparisons test
8A	6	In vivo	Ordinary One-way ANOVA	F (6, 35) = 4.906	P=0.001	**p< 0.01, ICH 1d: p = 0.0095, vs. Sham group	Šídák's multiple comparisons test
8C	6	In vivo	Unpaired t test	F (5, 5) = 6.093	P=0.004	**p< 0.01, ICH 1d: p = 0.0040 vs. Sham group, n = 6	-
8D	4	In vitro	Ordinary One-way ANOVA	F (5, 18) = 8.091	P=0.0004	**p< 0.01, OxyHb 6h: p = 0.0032 vs. Control group	Šídák's multiple comparisons test

* means p < 0.05; ** means p < 0.01

Figure	Test used	Stat-value
3B	Two-way ANOVA	Tubular: ICH vs. Sham: $p < 0.0001$; LV-shRNA-NC vs. LV-shRNA-MIC19: $p < 0.0001$; Vector vs. LV-MIC19: $p < 0.0001$<br>Short Tubular: ICH vs. Sham: $p = 0.0088$; LV-shRNA-NC vs. LV-shRNA-MIC19: $p = 0.0964$; Vector vs. LV-MIC19: $p = 0.7059$<br>Fragmented: ICH vs. Sham: $p = 0.6556$; LV-shRNA-NC vs. LV-shRNA-MIC19: $p = 0.0647$; Vector vs. LV-MIC19: $p = 0.8742$<br>Expanded: ICH vs. Sham: $p = 0.4478$; LV-shRNA-NC vs. LV-shRNA-MIC19: $p < 0.0001$; Vector vs. LV-MIC19: $p = 0.1960$<br>Large Spherical: ICH vs. Sham: $p = 0.5093$; LV-shRNA-NC vs. LV-shRNA-MIC19: $p < 0.0001$; Vector vs. LV-MIC19: $p = 0.0281$
4C	Two-way ANOVA	1d: ICH vs. Sham: $p < 0.0001$; LV-shRNA-NC vs. LV-shRNA-MIC19: $p > 0.9999$; Vector vs. LV-MIC19: $p = 0.273$<br>5d: ICH vs. Sham: $p < 0.0001$; LV-shRNA-NC vs. LV-shRNA-MIC19: $p = 0.9981$; Vector vs. LV-MIC19: $p < 0.0001$<br>7d: ICH vs. Sham: $p < 0.0001$; LV-shRNA-NC vs. LV-shRNA-MIC19: $p = 0.0953$; Vector vs. LV-MIC19: $p = 0.0025$<br>10d: ICH vs. Sham: $p < 0.0001$; LV-shRNA-NC vs. LV-shRNA-MIC19: $p = 0.4444$; Vector vs. LV-MIC19: $p < 0.0001$<br>14d: ICH vs. Sham: $p < 0.0001$; LV-shRNA-NC vs. LV-shRNA-MIC19: $p = 0.6747$; Vector vs. LV-MIC19: $p < 0.0001$<br>21d: ICH vs. Sham: $p < 0.0001$; LV-shRNA-NC vs. LV-shRNA-MIC19: $p > 0.9999$; Vector vs. LV-MIC19: $p = 0.0066$<br>28d: ICH vs. Sham: $p < 0.0001$; LV-shRNA-NC vs. LV-shRNA-MIC19: $p = 0.6918$; Vector vs. LV-MIC19: $p = 0.6918$
4D	Two-way ANOVA	1d: ICH vs. Sham: $p < 0.0001$; LV-shRNA-NC vs. LV-shRNA-MIC19: $p = 0.6205$; Vector vs. LV-MIC19: $p = 0.9973$<br>5d: ICH vs. Sham: $p < 0.0001$; LV-shRNA-NC vs. LV-

		shRNA-MIC19: $p > 0.9999$; Vector vs. LV-MIC19: $p = 0.8127$<br>7d: ICH vs. Sham: $p < 0.0001$; LV-shRNA-NC vs. LV-shRNA-MIC19: $p = 0.1190$; Vector vs. LV-MIC19: $p = 0.4484$<br>10d: ICH vs. Sham: $p < 0.0001$; LV-shRNA-NC vs. LV-shRNA-MIC19: $p = 0.3592$; Vector vs. LV-MIC19: $p = 0.4117$<br>14d: ICH vs. Sham: $p < 0.0001$; LV-shRNA-NC vs. LV-shRNA-MIC19: $p = 0.6014$; Vector vs. LV-MIC19: $p = 0.7974$<br>21d: ICH vs. Sham: $p < 0.0001$; LV-shRNA-NC vs. LV-shRNA-MIC19: $p > 0.9999$; Vector vs. LV-MIC19: $p = 0.0072$<br>28d: ICH vs. Sham: $p < 0.0001$; LV-shRNA-NC vs. LV-shRNA-MIC19: $p = 0.7312$; Vector vs. LV-MIC19: $p = 0.8127$
4G	Two-way ANOVA	29d: ICH vs. Sham: $p = 0.0020$; LV-shRNA-NC vs. LV-shRNA-MIC19: $p = 0.0331$; Vector vs. LV-MIC19: $p = 0.0231$<br>31d: ICH vs. Sham: $p = 0.0002$; LV-shRNA-NC vs. LV-shRNA-MIC19: $p = 0.0311$; Vector vs. LV-MIC19: $p = 0.0379$<br>33d: ICH vs. Sham: $p = 0.1391$; LV-shRNA-NC vs. LV-shRNA-MIC19: $p = 0.1909$; Vector vs. LV-MIC19: $p = 0.9419$
4H	Two-way ANOVA	29d: ICH vs. Sham: $p = 0.0002$; LV-shRNA-NC vs. LV-shRNA-MIC19: $p = 0.0138$; Vector vs. LV-MIC19: $p = 0.0123$<br>31d: ICH vs. Sham: $p < 0.0001$; LV-shRNA-NC vs. LV-shRNA-MIC19: $p = 0.0240$; Vector vs. LV-MIC19: $p = 0.0097$<br>33d: ICH vs. Sham: $p = 0.0215$; LV-shRNA-NC vs. LV-shRNA-MIC19: $p = 0.1614$; Vector vs. LV-MIC19: $p = 0.8092$
